# Caso 1/2020 – Coartação da Aorta Ístmica Muito Acentuada em Jovem com Hipertensão Arterial, Aliviada por Cateterismo Intervencionista

**DOI:** 10.36660/abc.20190484

**Published:** 2020-05-11

**Authors:** Edmar Atik, Raul Santiago Arrieta, Renata Cassar

**Affiliations:** 1 Clínica Particular Dr. Edmar Atik São PauloSP Brasil Clínica Particular Dr. Edmar Atik, São Paulo, SP – Brasil; 2 Hospital Sírio Libanês de São Paulo São PauloSP Brasil Hospital Sírio Libanês de São Paulo,São Paulo, SP – Brasil

**Keywords:** Cardiopatia Congênita/cirurgia, Coartação Aórtica/cirurgia, Estresse Psicológico, Hipertensão, Angioplastia com Balão/métodos, Stent

## Dados clínicos

Havia sido detectada hipertensão arterial há 6 meses após estresse estudantil em jovem de 16 anos de idade. Na ocasião imagens diagnósticas realizadas (ecocardiograma e angiotomografia) comprovaram a presença de coartação da aorta extrema na região ístmica, com muitas colaterais que preenchiam a aorta descendente. A pressão arterial de 170/80 mmHg diminuiu para níveis de 130 a 150/80 mmHg com o uso de propranolol-80 mg/dia. Havia sido operado anteriormente para fechamento de comunicação interatrial com 4 anos de idade. Referia cansaço aos esforços há alguns meses.

Exame físico: Bom estado geral, eupneico, acianótico, pulsos amplos nos membros superiores e ausentes nos inferiores. Peso: 45,5 Kg, Alt.: 163 cm, PAMSD e PAMSE = 155/80 mmHg, FC: 55 bpm. Aorta facilmente palpada na fúrcula.

Precórdio: *ictus cordis* não palpado e sem impulsões sistólicas na borda esternal esquerda. Bulhas cardíacas normofonéticas, sopro sistólico rude, ++/4 na fúrcula e faces laterais do pescoço e sopro diastólico suave e aspirativo, +++/4, na borda esternal esquerda. Não eram audíveis sopros no dorso do tórax. Fígado não palpado e pulmões limpos.

## Exames complementares

**Eletrocardiograma:** Ritmo sinusal, sinais de sobrecarga de ventrículo esquerdo com índice de Sokollof de 46 mm e com repolarização ventricular normal. AP = +40^o^, AQRS = +60^o^, AT = +30^o^.

**Figura 1:**
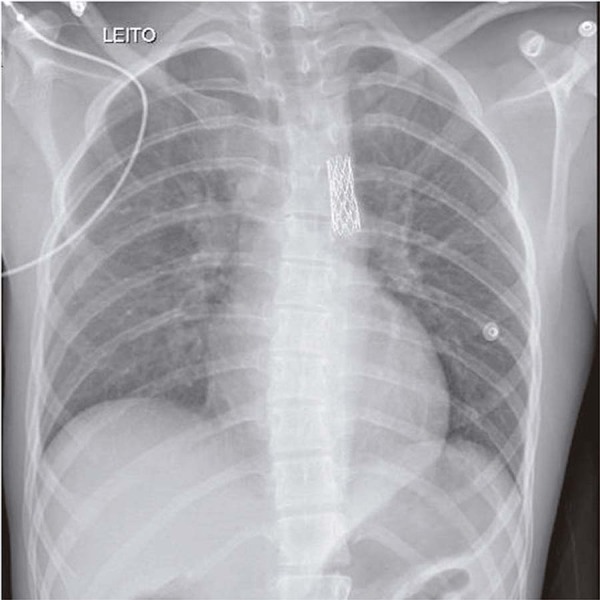
- Radiografia de tórax em PA salienta área cardíaca normal com índice cardiotorácico de 0,50 e trama vascular pulmonar normal. Colocação de stent no início da aorta descendente salienta a dilatação pós-estenótica da aorta.

**Radiografia de tórax:** Área cardíaca normal (índice cardiotorácico = 0,50). Pedículo vascular alto mostra imagem em três (3) com maior dilatação na parte inferior orientando ao diagnóstico da coartação da aorta nessa região. Havia sinais de corrosão costal à direita ( Figura 1 ).

**Ecocardiograma:** Salienta cavidades cardíacas normais e sem hipertrofia miocárdica. Gradiente máximo de 14,7 e médio de 6,8 mmHg na valva aórtica. As dimensões eram: Ao = 27, AE = 28, VE = 47, septo = 9, FE-VE = 68%, PS-VD = 28 mmHg.

**Angiotomografia:** Coartação da aorta após a emergência da artéria subclávia esquerda com circulação colateral evidente e pronunciada. Aorta ascendente= 28 mm, aorta descendente após a CoAo = 21 mm e a aorta tóraco-abdominal = 14 mm.

**Monitorização ambulatorial da** **pressão arterial (MAPA)** : Pressão arterial máxima = 170/100 mmHg e na maioria do tempo = 130-140/60-70 mmHg.

**Holter:** Extrassístoles ventriculares em número de 2.315 (3%) dentre 77 166 batimentos.

**Diagnóstico clínico:** Coartação da aorta acentuada na região ístmica com circulação colateral exuberante e valva aórtica bivalvular em evolução natural de jovem com hipertensão arterial.

**Raciocínio clínico:** Os elementos diagnósticos da coartação da aorta eram evidentes, representados pela ausência dos pulsos arteriais nos membros inferiores, a hipertensão arterial nos membros superiores, acompanhados do sopro sistólico na fúrcula, e da sobrecarga de ventrículo esquerdo no eletrocardiograma, além da imagem em três (3) na radiografia de tórax. A confirmação diagnóstica facilmente foi estabelecida pelas imagens do ecocardiograma e da angiotomografia.

**Diagnóstico diferencial** : A coartação da aorta congênita deve ser distinguida de anomalias adquiridas que também causam obstrução em vários níveis da aorta, como na doença de Takayassu.

**Conduta:** Dos dois caminhos para a correção da coartação da aorta, o cirúrgico^1^ e o percutâneo,^2^ houve opção pelo último. Antes, foi realizado o cateterismo cardíaco que revelou pressão na aorta ascendente de 150/80 com média de 96 mmHg e na aorta descendente de 50/30 e 40 mmHg de média. A angiografia salientava estreitamento progressivamente maior a partir da artéria subclávia esquerda, cujo diâmetro de 12 mm era o mesmo do da croça da aorta, até cerca de 40 mm abaixo, quando então se tornava puntiforme com orifício máximo de 2 mm e dilatação pós estenótica de 18 mm de diâmetro. Havia grande circulação colateral.

**Figura 2:**
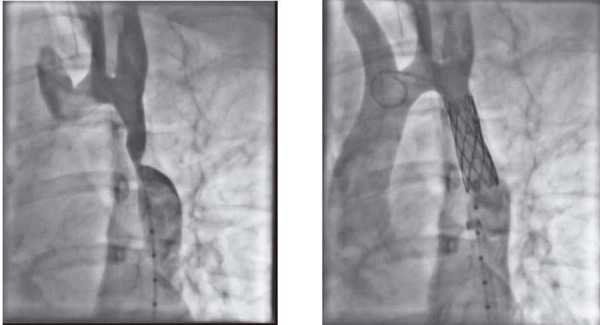
- Angiografia cardíaca mostra a acentuada coartação da aorta, cerca de 40 mm após a emergência da artéria subclávia esquerda, na imagem a esquerda e a ampla dilatação dessa região após a colocação de stent, na imagem à direita.

Com esse quadro, foi realizada a pré-dilatação com *balão Mustang (Boston-* 5/20 mm) da região ístmica com coartação. Nova angiografia mostrou aumento do diâmetro da aorta com coartação, sem sinais de dissecção ou de aneurisma. Através de *Bainha tipo Mullins* 14 fr, foi posicionado e implantado *stent CP* recoberto e montado em balão 14/40 mm (BIB balloon). Novas angiografias evidenciaram nítida melhora da coartação da aorta ( Figura 2 ). As pressões posteriores se equivaleram na aorta ascendente e descendente em 127/67 e média de 87 mmHg.

**Comentários:** A coartação da aorta mesmo acentuada pode evoluir a longo prazo sem alterações significativas desde que haja o desenvolvimento de circulação colateral que minimize a obstrução aórtica. Esse pensamento vem de encontro com a evolução observada neste caso sem que tivesse desenvolvido ao menos hipertrofia miocárdica ou algum grau de disfunção miocárdica. Outro aspecto que chama a atenção neste caso clínico foi o diagnóstico tardio da anomalia, quando incidentalmente foi observado uma elevada pressão arterial nos membros superiores. Tal fato expressa que o exame clínico prévio deste paciente não havia sido certamente executado com os requintes de uma semiologia mais adequada. O procedimento percutâneo tem se tornado o mais indicado na coartação da aorta, principalmente no jovem e no adulto, por menos complicações e com efetividade semelhante à do procedimento cirúrgico.^1 , 2^ As imagens angiográficas atestam bem essa assertiva.
