# Supplementary material for: Caso 1/2020 – Coartação da Aorta Ístmica Muito Acentuada em Jovem com Hipertensão Arterial, Aliviada por Cateterismo Intervencionista
Source: Arq Bras Cardiol. 2020 May 11;114(4 Suppl 1):38–9. [Article in Portuguese] doi: 10.36660/abc.20190484 (PMC8149106; doi:10.36660/abc.20190484)
Supplement: Translation

# Translation: Case 1/2020 – Very Accentuated Isthmic Coarctation of the Aorta in a Young Individual with Arterial Hypertension Relieved by Interventional Catheterization

**Authors:** Edmar Atik, Raul Santiago Arrieta, Renata Cassar

**Affiliations:** 1 Brazil Clínica Particular Dr. Edmar Atik, São Paulo, SP – Brazil; 2 Brazil Hospital Sírio Libanês de São Paulo, São Paulo, SP – Brazil

**Keywords:** Heart Defects Congenital/surgery, Aortic, Coarctation/surgery, Stress Psychological, Hypertension: Angioplasty, Balloon/methods, Stent

## Clinical data

Arterial hypertension had been detected 6 months before, after study-related stress in a 16-year-old individual. At the time, diagnostic images (echocardiography and angiotomography) confirmed the presence of accentuated isthmic coarctation of the aorta, with many collaterals that filled the descending aorta. Blood pressure was 170/80 mmHg, which decreased to 130 to 150/80 mmHg with propranolol-80 mg/day. He had been previously submitted to surgery for atrial septal defect closure at 4 years of age. He reported fatigue at exertion since a few months before.

Physical examination: Good overall status, eupneic, acyanotic, wide pulses in the upper limbs and absent in the lower limbs. Weight: 45.5 Kg, Height: 163 cm, right upper limb BP and left upper limb BP = 155/80 mmHg, HR: 55 bpm. Aorta easily palpated at the suprasternal notch.

Precordium: non-palpable apex beat and no systolic impulses along the left sternal border. Normal heart sounds, rough systolic murmur, ++/4 in the suprasternal notch and lateral neck surfaces, and mild and aspirating diastolic murmur, +++/4, in the left sternal border. There were no audible murmurs on the back of the thorax. The liver was not palpated, and the lungs were clear.

## Complementary examinations

**Electrocardiogram:** Sinus rhythm, signs of left ventricular overload with Sokoloff index of 46 mm and normal ventricular repolarization. AP = +40^o^, AQRS = +60^o^, AT = +30^o^.

**Chest x-ray:** Normal cardiac area (cardiothoracic index = 0.50). High vascular pedicle shows a three (3)-shaped image with greater dilation in the lower part, leading to the diagnosis of coarctation of the aorta (CoAo) in this region. There were signs of costal corrosion on the right ( Figure 1 ).

**Figure 1:** - PA Chest X-ray highlights normal cardiac area with a cardiothoracic index of 0.50 and normal pulmonary vascular network. Stent placement at the beginning of the descending aorta highlights post-stenotic dilation of the aorta.

**Echocardiography:** It showed normal heart chambers without myocardial hypertrophy. Maximum gradient 14.7 and mean 6.8 mmHg in the aortic valve. The dimensions were: Ao = 27, LA = 28, LV = 47, septum = 9, LVEF = 68%, RVSP = 28 mmHg.

**Angiotomography:** Coarctation of the aorta after emergence of the left subclavian artery with pronounced and marked collateral circulation. Ascending aorta = 28 mm, descending aorta after CoAo = 21 mm and thoracoabdominal aorta = 14 mm.

**Ambulatory Blood Pressure Monitoring (ABPM):** Maximum blood pressure = 170/100 mmHg and most of the time = 130-140/60-70 mmHg.

**Holter:** Ventricular extrasystoles: 2,315 (3%) of 77,166 beats.

**Clinical diagnosis:** Accentuated coarctation of the aorta in the isthmus with exuberant collateral circulation and bivalvular aortic valve undergoing natural evolution in young individual with arterial hypertension.

**Clinical reasoning:** The diagnostic elements of coarctation of the aorta were evident, represented by the absence of arterial pulses in the lower limbs, arterial hypertension in the upper limbs, accompanied by systolic murmur in the suprasternal notch, and left ventricular overload on the electrocardiogram, in addition to the three (3)-shaped image on chest X-ray. Diagnostic confirmation was easily established by the echocardiogram and angiotomography images.

**Differential diagnosis** : Congenital coarctation of the aorta should be differentiated from acquired anomalies that also cause obstruction at several levels of the aorta, such as Takayasu disease.

**Conduct:** Of the two approaches for correction of aortic coarctation, the surgical^1^ and the percutaneous,^2^ the latter was chosen. Previously, cardiac catheterization was performed, which disclosed pressure in the ascending aorta of 150/80 with a mean of 96 mmHg and in the descending aorta of 50/30 and mean of 40 mmHg. The angiography showed progressively greater narrowing from the left subclavian artery, of which diameter of 12 mm was the same as that of the aortic arch, up to about 40 mm below, when it then became punctiform with a maximum orifice of 2 mm and post-stenotic dilation with 18 mm in diameter. There was large collateral circulation.

Considering this picture, a pre-dilation with Mustang balloon (Boston-5/20 mm) was performed of the isthmus region with coarctation. A new angiography showed increased diameter of the aorta with coarctation without signs of dissection or aneurysm. Using a 14 fr Mullins sheath, a covered 14/40 mm CP stent (BIB balloon) was positioned and implanted. New angiographies showed a clear improvement in aortic coarctation ( Figure 2 ). Posterior pressures were equivalent in the ascending and descending aorta at 127/67 and mean of 87 mmHg.

**Figure 2:** - Cardiac angiography shows the accentuated coarctation of the aorta, approximately 40 mm after the emergence of the left subclavian artery in the image on the left and the wide dilation of this region after stent placement in the image on the right.

**Comments:** Coarctation of the aorta, even when accentuated, can have a long-term evolution without significant changes, as long as collateral circulation develops to minimize aortic obstruction. This thought is in opposition with the evolution observed in this case, which did not develop even myocardial hypertrophy or some degree of myocardial dysfunction. Another aspect that draws attention in this clinical case was the late diagnosis of the anomaly, when a high blood pressure in the upper limbs was incidentally observed. This fact shows that the previous clinical examination of this patient had certainly not been performed with the refinements of a more adequate semiology. The percutaneous procedure has become the most indicated in the coarctation of the aorta, especially in young individuals and adults, due to fewer complications and similar effectiveness to that of the surgical procedure.^1 , 2^ Angiographic images demonstrate this assertion.
